# The Method of Dispensing Calcium Chloride

**Published:** 1907-06-01

**Authors:** 


### THE METHOD OF DISPENSING CALCIUM CHLORIDE.

Calcium chloride is a medicine which is finding more and more favour with the profession. In suitable doses it is known to increase the force of the heart beat, to increase the coagulability of the blood, and to augment the healing powers of the tissues. It is therefore largely used as a prophylactic against heart failure in febrile conditions such as lobar pneumonia and typhoid fever; as a curative agent in hæmophilia, scurvy, purpura, and in many conditions in which spontaneous bleedings are liable to occur; and in such affections as indolent ulcers, chilblains, and other troubles dependent upon a sluggish circulation. There is every likelihood that it will be prescribed for so many different lesions that some of them will not be benefited by it; and presently it will fall into disrepute, notwithstanding its undoubted value when properly used.

### The Prescription.

There is, however, another important reason why its prescription is liable to meet with disfavour—on the part of the patient—and that is its vile taste. It is not only salt, but also to some degree bitter, and, moreover, nauseous. The same applies to calcium iodide, or to a mixture of calcium chloride and potassium iodide. When it is desired to use the last two together the greatest care must be taken in composing the prescription. Liquid extract of liquorice is a common aid in covering the taste of nauseous drugs, but it produces a hopeless yellow soup when mixed with calcium chloride and potassium iodide. It will be found that the following mixture, though not absolutely pleasant, can be taken without nausea:—

| | | |
|---|---|---|
| ℞ | Calcii chloridi ... ... ... ... | gr. xv. |
| | Potassii iodidi ... ... ... ... | gr. v. |
| | Syrupus limonis ... ... ... | ʒiv. |
| | Aquam chloroformi ad ... ... | ℥j. |

Misce fiat haustus.

Sig. Two tablespoonfuls of the medicine to be diluted with an equal quantity of effervescing water, such as soda water, and taken as often as directed.

It will be found that at least 4 drachms of lemon syrup are needed in each dose to cover the salt taste of the halogens, and that the medicine is then a little too sweet unless it is diluted with the soda water. The chloride of calcium is deliquescent, so that it cannot be kept as a powder except in sealed bottles or tins; for the same reason it cannot be dispensed in cachets. Capsules can be employed by druggists, but they are not conveniently made by a doctor practising in the country.

